# Effects of a carbohydrate-restricted diet on emerging plasma markers for cardiovascular disease

**DOI:** 10.1186/1743-7075-3-19

**Published:** 2006-05-04

**Authors:** Richard J Wood, Jeff S Volek, Steven R Davis, Carly Dell'Ova, Maria Luz Fernandez

**Affiliations:** 1 Department of Nutritional Sciences, University of Connecticut, Storrs, CT, USA; 2 Human Performance Laboratory, Department of Kinesiology, University of Connecticut, Storrs, CT, USA

## Abstract

**Background:**

Increasing evidence supports carbohydrate restricted diets (CRD) for weight loss and improvement in traditional markers for cardiovascular disease (CVD); less is known regarding emerging CVD risk factors. We previously reported that a weight loss intervention based on a CRD (% carbohydrate:fat:protein = 13:60:27) led to a mean weight loss of 7.5 kg and a 20% reduction of abdominal fat in 29 overweight men. This group showed reduction in plasma LDL-cholesterol and triglycerides and elevations in HDL-cholesterol as well as reductions in large and medium VLDL particles and increases in LDL particle size. In this study we report on the effect of this intervention with and without fiber supplementation on plasma homocysteine, lipoprotein (a) [Lp(a)], C-reactive protein (CRP), interleukin-6 (IL-6), and tumor necrosis factor alpha (TNF-α).

**Methods:**

Twenty nine overweight men [body mass index (BMI) 25–35 kg/m^2^] aged 20–69 years consumed an ad libitum CRD (% carbohydrate:fat:protein = 13:60:27) including a standard multivitamin every other day for 12 wk. Subjects were matched by age and BMI and randomly assigned to consume 3 g/d of either a soluble fiber supplement (n = 14) or placebo (n = 15).

**Results:**

There were no group or interaction (fiber × time) main effects, but significant time effects were observed for several variables. Energy intake was spontaneously reduced (-30.5%). This was accompanied by an increase in protein intake (96.2 ± 29.8 g/d to 107.3 ± 29.7 g/d) and methionine intake (2.25 ± 0.7 g/d, to 2.71 ± 0.78 g/d; *P *< 0.001). Trans fatty acid intake was significantly reduced (-38.6%) while dietary folate was unchanged, as was plasma homocysteine. Bodyweight (-7.5 ± 2.5 kg) was reduced as was plasma Lp(a) (-11.3%). Changes in plasma Lp(a) correlated with reductions in LDL-cholesterol (r = .436, *P *< 0.05) and fat loss (r = .385, *P *< 0,05). At wk 12, both CRP (-8.1%) and TNF-α (-9.3%) were reduced (*P *< 0.05) independently of weight loss. IL-6 concentrations were unchanged.

**Conclusion:**

A diet based on restricting carbohydrates leads to spontaneous caloric reduction and subsequent improvement in emerging markers of CVD in overweight/obese men who are otherwise healthy.

## Background

Evidence continues to mount supporting the use of carbohydrate restricted diets (CRD) for weight loss and improvement in cardiovascular risk [1]. In particular, CRD have been repeatedly shown to promote weight loss and improve fasting and postprandial triglycerides, and plasma HDL-cholesterol (HDL-C), which indicates that such an approach may be an important treatment option for the dyslipidemia associated with the metabolic syndrome [2]. Also, CRD appear to be particularly effective in reducing intra-abdominal fat, which is known to correlate with numerous cardiovascular risk factors [3]. Though traditional markers of cardiovascular risk are still an integral part of initial cardiovascular risk assessment, several additional risk factors have emerged that provide a more complete picture of total risk status.

Elevated plasma levels of the cholesteryl-ester rich lipoprotein (a) [Lp(a)] increase the risk for development of cardiovascular disease through both proatherogenic and prothrombotic properties [4]. Plasma Lp(a) is of particular interest because of reports that levels are strongly controlled by genetics, and it is generally considered that standard diet and exercise therapy known to alter plasma levels of other lipoproteins have little effect on Lp(a) [5], although intake of dietary trans fatty acids have been reported to increase concentrations of Lp (a) [6], [7]. However, dietary interventions in which Lp(a) levels were examined appear to be largely restricted to low-fat diets.

An elevated concentration of homocysteine in plasma is an independent risk factor for cardiovascular disease that is treatable through dietary and other interventions [8,9]. Insufficient vitamin B 12 and folate correlate with plasma homocysteine levels, and supplemental B vitamins and folate can be part of an effective dietary treatment [4]. However, it is not clear that treatment of hyperhomocysteinemia will reduce cardiovascular disease risk [10]. Consuming a low carbohydrate, high protein diet without vitamin supplementation has been reported to lead to elevated plasma homocysteine [11].

Inflammatory processes play an integral part in the development and exacerbation of atherosclerotic lesions. Having an elevated level of the acute-phase reactant C-reactive protein (CRP) is considered a strong, independent predictor of coronary heart disease [12], and is believed to play a direct role in the development of atherosclerotic plaque through effects on the vascular endothelium [13]. A key precursor to the hepatic production of CRP is interleukin 6 (IL-6). Plasma IL-6 levels can predict the development of diabetes [14], and is thought to be involved in the pathogenesis of atherosclerotic vascular disease [15]. Another inflammatory marker involved in both the generation and propagation of atherosclerosis is tumor necrosis factor α (TNF-α). TNF-α is thought to be responsible for endothelial dysfunction [16], thus could also play a role in atherosclerosis. Most importantly, each of these inflammatory markers are elevated in obesity, and can be produced by adipose tissue [16,17], which underscores the importance of weight loss in cardiovascular risk management.

The purpose of this diet study was to determine the effects of a 12-wk CRD with and without soluble fiber supplementation in overweight men on plasma levels of homocysteine, Lp(a), CRP, IL-6, and TNF-α. Our hypothesis was that CRD would improve these emerging risk factors for CVD.

## Methods

### Study design

As previously described [18], the study was designed to investigate the effects of adding a soluble fiber supplement to a CRD on clinical markers of cardiovascular risk including plasma lipids, glucose, blood pressure, and body composition. A 12-wk placebo-controlled, double blind, parallel arm design was used. Subjects were carefully matched according to BMI and age, then randomly assigned to supplement a CRD with 3 g/d of either Konjac-mannan (n = 15) or a placebo (n = 15) containing maltodextrin and free from Konjac-mannan.

### Subjects

A total of 30 men with a BMI between 25 and 35 kg/m^2 ^aged 20–69 years volunteered to participate in the study. One subject from the fiber group left the study due to military obligations. Subjects completed a detailed medical history questionnaire during subject recruitment. Exclusion criteria were consumption of a CRD (including any form of purposeful carbohydrate restriction such as the Atkins diet, South Beach diet, Sugar Busters, and the Zone diet) or weight loss greater than 2.5 kg in the past 6 months, presence of cardiovascular or thyroid disease or diabetes mellitus, consumption of lipid lowering prescriptions or supplements, or blood pressure greater than 160/90 mm Hg. At baseline, subject characteristics were: age, 38.8 ± 14.2 years; body weight, 93.3 ± 14.0 kg; body fat 32.1 ± 4.4%; and BMI 29.7 ± 3.5 kg/m^2^. All procedures were approved by the Institutional Review Board at the University of Connecticut, and all subjects provided written informed consent to participate.

### Diet

A detailed description of the experimental diet has been reported previously [18]. In brief, this was a free-living study and no food was provided to subjects. Registered dietitians held group meetings and instructed subjects how to follow a CRD and how to accurately complete weighed food records. Each subject received written materials to reinforce the principles covered during the meeting. A five-day weighed food record was completed prior to the intervention and seven-day weighed food records were completed at weeks 1, 6 and 12. Diet data presented in the current study is from baseline and wk 12. The diet was specifically designed to restrict carbohydrates so that subjects produced ketones detectable in urine. Our previous work indicates that this requires a carbohydrate intake of about 10% of total energy. The diet was *ad libitum *and no guidelines were given regarding energy consumption. Subjects were provided with a generic multivitamin/mineral to be consumed every-other day. Each tablet provided the following quantities of select micronutrients: riboflavin 1.7 mg/d; niacin 20 mg/d; B6 2 mg/d; B12 6 μg/d; and folic acid 400 μg/d. At baseline, subjects were sedentary to mildly active, and were instructed to maintain baseline levels of physical activity throughout the intervention.

### Data collection

At baseline and wk 12, subjects reported to the laboratory after an overnight fast. Whole blood was obtained from an antecubital vein into EDTA tubes, and plasma was obtained via centrifugation at 1500 × g at 4°C for 20 min. After plasma was isolated, a preservation cocktail was added to the samples (5 mL/L aprotinin, 1 mL/L PMSF and 1 mL/L sodium azide). Plasma was stored in individual aliquots at -80°C for later analysis. Plasma levels of homocysteine, Lp(a), CRP, TNF-α, and IL-6 were determined for baseline and wk 12.

### Dietary assessment

Diet records were analyzed using the Nutrition Data System 5.0 (Minneapolis, MN). Analyses were completed to determine the mean daily intake of kcal and energy contribution from fat, carbohydrate, and protein. Also, mean daily intake of total folate, and vitamins riboflavin, niacin, B6, and B12 were determined.

### Anthropometrics

Bodyweight was determined on the same calibrated digital scale to the nearest 100 g with subjects in light clothing and not wearing shoes. Body composition was determined using dual-energy X-ray absorptiometry (Prodigy™, Lunar Corporation, Madison, WI).

### Determination of plasma variables

#### Homocysteine, cysteine, cysteinylglycine

Concentrations of homocysteine, cysteine, and cysteinylglycine in plasma were measured by high pressure liquid chromatography with fluorometric detection [19] on a Beckman HPLC system (Fullerton, CA) with refrigerated autosampler and Jasco FP2020 fluorescence detector (Tokyo, Japan).

#### Lp(a)

Plasma Lp(a) was determined in duplicate using a sandwich ELISA (#1740–9; Diagnostic Automation, Inc., Calabasas, CA, USA) with a dynamic range of 1.0–165 mg/dL. The intra-assay coefficient of variation (CV) was 9.1%. Absorbance was determined using a VersaMax tunable microplate reader with SoftMax^® ^Pro data reduction software (Molecular Devices, Sunnyvale, CA, USA).

#### CRP, IL-6, TNF-α

High-sensitivity CRP was determined in duplicate using a sandwich ELISA (#30–9710 s; American Laboratory Products Company, Windham, NH, USA). The sensitivity of the hsCRP assay was 0.124 ng/mL, and the intra-assay CV was 7.3%. Plasma IL-6 and TNF-α were determined in duplicate using a high-sensitivity ELISA (#HS600B for hsIL-6, and #HSTA00C for hsTNF-α; R&D Systems, Minneapolis, MN, USA). For the hsIL-6 assay the sensitivity was 0.039 pg/mL, and the intra-assay CV was 5.2%. For the hsTNF-α assay the sensitivity was 0.12 pg/mL and the intra-assay CV was 8.9%. Absorbances were determined using a VersaMax tunable microplate reader with SoftMax^® ^Pro data reduction software (Molecular Devices, Sunnyvale, CA, USA).

### Statistical analyses

Dependent variables were analyzed using a 2 × 2 analysis of variance with supplement assignment (Fiber and Placebo) as the between-factor and time (Wk 0 and 12) as the within factor. Pearson correlation coefficients were used to determine the relationship between changes in dependent variables. Differences with a *P *< 0.05 were considered significant. Data are represented as means ± standard deviation. Statistical analyses were completed using SPSS version 12.0 for Windows.

## Results

### Dietary consumption, bodyweight, and compliance

**Figure 1:**
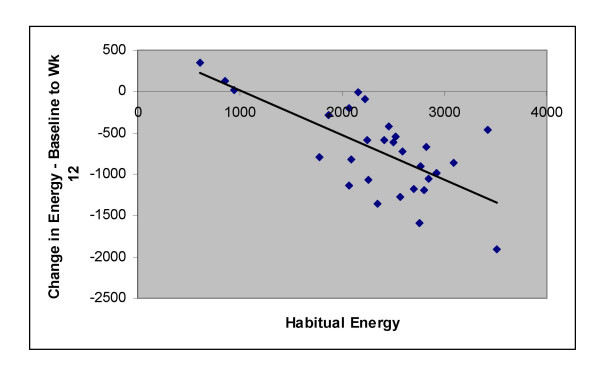
**Baseline (habitual) energy intake (kcal) vs. change in energy intake from baseline to week 12**. Mean kcal reduction from baseline to week 12 (n = 29) was 718 kcal/day. Overweight men consumed a CRD for 12 weeks; r = -.687; *P *< 0.01.

Compared to the habitual diet, the introduction of a CRD lead to a decrease in caloric consumption (*P *< 0.001) (Table 1) as well as carbohydrate consumption (-73.9%; *P *< 0.001), while protein consumption increased (11.5%; *P *< 0.05), and fat intake was unchanged at week 12 (4.3%; *P *> 0.05). Saturated fat intake was also unchanged (*P *> 0.05). Habitual energy intake vs. change in energy intake from baseline to week 12 is plotted in Figure 1. Energy intake was spontaneously reduced by 718 ± 530 kcal per day, characterized by a significant reduction in carbohydrate intake (-200 ± 64 g/d; *P *< 0.001) and significant increase in protein intake (11 ± 27 g/d; *P *< 0.05), but no change in fat intake (4 ± 41 g/d; *P *> 0.05). Thirteen subjects reduced fat intake during the intervention. As expected by the increase in protein intake, methionine intake increased significantly. Total folate and vitamin B 12 intake were unchanged (*P *> 0.05), but vitamin B 6 intake increased (*P *< 0.001) in comparison to baseline. Also, by week 12 trans-fat intake was reduced (-37.7%; *P *< 0.001). Both bodyweight (-7.5 ± 2.5 kg) and fat mass (-5.7 ± 2.6 kg) were significantly reduced. There were no significant differences between the fiber and placebo groups for any diet or anthropometric measure. Compliance to the CRD was high throughout the intervention as determined through the presence of urinary ketones (data not shown) and dietary logs.

**Table 1:** Changes dietary macro- and micronutrients between habitual and intervention (CRD) diets for 12 weeks in overweight men.

Nutrient	Baseline (Habitual)	Week 12 of CRD	*P *Value (Time)
Total Energy (kcal)	2350 ± 676	1632 ± 496	< 0.001
Saturated Fat (g)	36.4 ± 16.2	36.8 ± 15.1	> 0.05
MUFA (g)	39.2 ± 15.9	45.2 ± 17.2	> 0.05
PUFA (g)	19.0 ± 7.2	17.9 ± 8.1	> 0.05
Trans Fats (g)	6.1 ± 2.7	3.8 ± 1.8	< 0.001
Methionine (g)	2.2 ± 0.7	2.7 ± 0.8	< 0.01
Total Folate (μg)	434.6 ± 174.1	455.0 ± 121.1	> 0.05
Vitamin B6 (mg)	2.0 ± 1.0	2.7 ± 0.6	< 0.001
Vitamin B12 (μg)	10.1 ± 16.5	11.2 ± 5.3	> 0.05

Data are presented as means ± SD (n = 29). Data were analyzed using a Student's *t *test. *P *values refer to the difference between baseline and week 12. Subjects consumed a CRD supplemented by standard multivitamin consumed every-other day for 12 weeks. MUFA = monounsaturated fatty acids; PUFA = polyunsaturated fatty acids. Analysis was performed on 5-day weighed food records for baseline and 7 day weighed food records for week 12.

### Plasma homocysteine, cysteine, and cysteinylglycine

Despite increased methionine and unchanged total folate intake, plasma homocysteine was not significantly increased (Table 2). Furthermore, plasma cysteine and cysteinylclycine were also unchanged (*P *> 0.05). No significant differences between the fiber and placebo groups existed.

**Table 2:** Changes in plasma levels of homocysteine, cysteine, and cysteinylglycine from baseline to week 12 in overweight men who consumed a CRD.

Parameter	Baseline	Week 12	*P *Value (Time)
Homocysteine (μmol/L)			
Fiber	8.1 ± 1.2	8.6 ± 2.2	> 0.05
Placebo	8.1 ± 1.4	8.3 ± 1.5	> 0.05
Cysteine (μmol/L)			
Fiber	264.4 ± 34.1	258.9 ± 45.3	> 0.05
Placebo	269.0 ± 30.5	266.2 ± 40.7	> 0.05
Cysteinylglycine (μmol/L)			
Fiber	30.3 ± 5.1	29.9 ± 4.5	> 0.05
Placebo	30.9 ± 3.8	29.0 ± 5.5	> 0.05

Data are presented as means ± SD for the fiber group (n = 14) and the placebo group (n = 14). *P *values refer to the difference between baseline and week 12. No significant time or treatment effects were observed in any measured plasma marker of homocysteine metabolism. Subjects supplemented the CRD by consuming a standard multivitamin every-other day.

### Plasma lipids and Lp(a)

**Figure 2:**
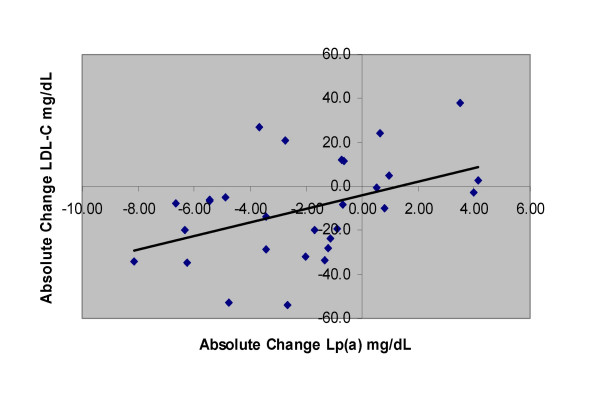
**Relationship between absolute change in Lp(a) and LDL-C for overweight men who followed a 12-week CRD for weight loss**. Changes in Lp(a) and LDL-C are in mg/dL. Subjects were overweight and slightly obese men (n = 29). Calculations using a Pearson correlation coefficient indicated a relationship (r = .436, *P *< 0.05).

**Figure 3:**
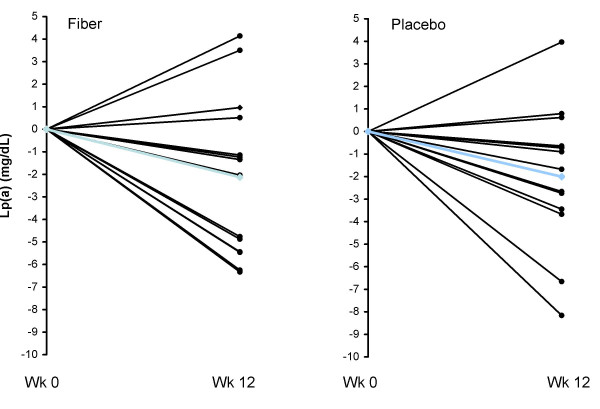
**Individual responses of Lp(a) for the fiber (left panel) and placebo (right panel) groups from baseline to week 12 for overweight men (n = 29) who consumed a CRD**. Blue line represents the mean. Lp(a) was significantly reduced over time (*P *< 0.05) with no treatment effects.

Mean relative changes for triglycerides, HDL-C, and LDL cholesterol (LDL-C) for all subjects have been reported previously, and were -34.5%, 12.6%, and -7.0% respectively (*P *< 0.05) [18]. Plasma Lp(a) was significantly reduced (-11.7%) from 17.9 ± 10.3 mg/dL at baseline to 15.8 ± 9.2 mg/dL at wk 12. Reductions in plasma Lp(a) were correlated with reductions in LDL-C (r = .436, *P *< 0.05; Figure 2) and fat mass (r = .385, *P *< 0.05), but not reduction in bodyweight (r = .280, *P *> 0.05). There were no differences between the fiber and placebo groups in plasma lipids. Twenty-two of 29 subjects had reductions in plasma Lp(a) at wk 12. Individual changes in Lp(a) from baseline to week 12 are depicted in Figure 3.

### Plasma CRP, IL-6, and TNF-α

**Figure 4:**
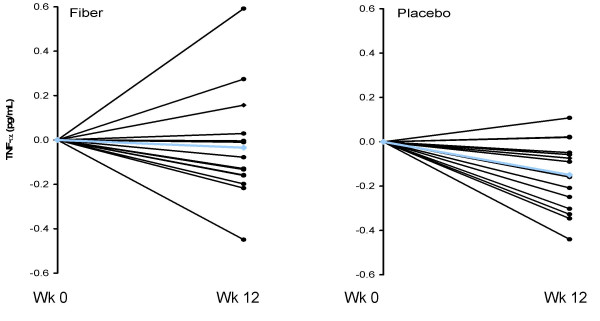
**Individual responses of TNF-alpha for the fiber (left panel) and placebo (right panel) groups from baseline to week 12 for overweight men (n = 29) who consumed a CRD**. Blue line represents the mean. TNF-alpha was significantly reduced over time (*P *< 0.05) with no treatment effect.

**Figure 5:**
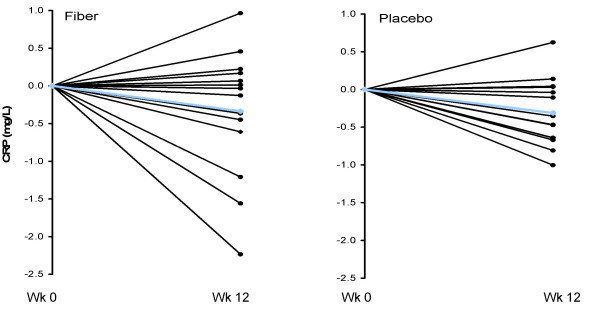
**Individual responses of CRP for the fiber (left panel) and placebo (right panel) groups from baseline to week 12 for overweight men (n = 29) who consumed a CRD**. Blue line represents the mean. CRP was significantly reduced over time (*P *< 0.05) with no treatment effect.

**Figure 6:**
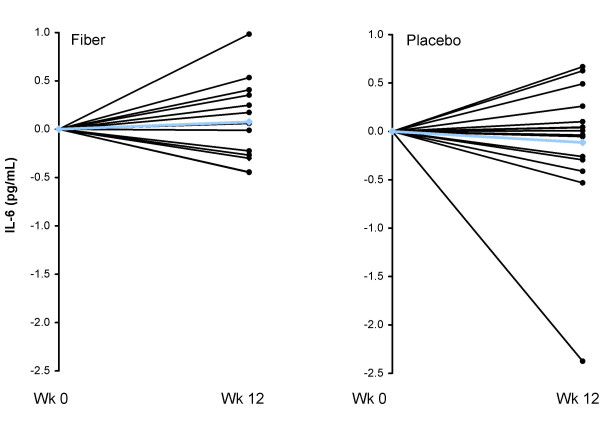
**Individual responses of IL-6 for the fiber (left panel) and placebo (right panel) groups from baseline to week 12 for overweight men (n = 29) who consumed a CRD**. Blue line represents the mean. There was no significant time or treatment effect for IL-6 (*P *> 0.05).

Consumption of the CRD and subsequent weight loss led to significant reductions in hsCRP, and hsTNF-α, but unchanged hsIL-6, with no significant differences between groups (Table 3). Individual changes in hsTNF-α, hsCRP, and hsIL-6 for both the fiber and placebo groups are shown in Figures 4, 5, and 6, respectively. Changes in hsCRP and hsTNF-α were not correlated with change in bodyweight or fat mass (*P *> 0.05).

**Table 3:** Response of plasma markers of inflammation to a CRD in overweight men.

Inflammatory Marker	Baseline	Week 12	*P *Value
hsCRP (mg/L)			
Fiber	1.68 ± 1.50	1.35 ± 0.95	< 0.05
Placebo	1.86 ± 1.29	1.55 ± 1.23	< 0.05
hsIL-6 (pg/mL)			
Fiber	1.31 ± 0.39	1.39 ± 0.50	> 0.05
Placebo	2.00 ± 1.62	1.88 ± 1.07	> 0.05
hsTNF-α (pg/mL)			
Fiber	1.29 ± 0.26	1.25 ± 0.30	< 0.05
Placebo	1.18 ± 0.27	1.03 ± 0.27	< 0.05

Data are presented as means ± SD for the fiber group (n = 14) and the placebo group (n = 14). Subjects consumed a CRD supplemented with a standard multivitamin consumed every-other day for 12 weeks. *P *values are for changes over time. No significant treatment effects existed.

## Discussion

The purpose of this study was to report observations about changes in emerging risk factors following weight loss induced by a CRD with or without soluble fiber supplementation in overweight and slightly obese men. Though comparisons of these changes cannot be made to other forms of diet therapy, these data contribute to the small amount of evidence currently available about the effects of CRD on markers of CVD risk not traditionally measured. The main findings of the study were that a CRD resulted in a spontaneous reduction in caloric intake in 93% of subjects and favorable effects on a number of non traditional biomarkers including a significant reduction in plasma Lp(a) in 76% of subjects and a significant reduction in the inflammatory markers CRP in 69% and TNF-α in 76% of subjects. There were no effects of adding additional soluble fiber to a CRD on these responses. Though dietary fiber has been shown to reduce CVD risk, it appears that the significant weight loss in our subjects has overshadowed any potential benefits from the fiber supplementation.

Consistent with other reports [20-22], simply instructing subjects to restrict carbohydrates resulted in a spontaneous reduction in energy intake (mean 718 kcal/day), suggesting that a CRD may be easier to adhere to than diet therapies that specifically target energy restriction. The reduction in caloric intake was predominantly due to the 74% reduction in carbohydrate. There is a common belief that CRD are high in fat, but in this study the absolute amount of total fat and saturated fat intake was unchanged and protein intake only modestly increased.

### Homocysteine

Weight loss can lead to increases in homocysteine concentrations [23,24], and alter the dose-response relationship between both serum folate and vitamin B-12 levels (i.e., higher serum folate and B-12 levels are required to maintain the same homocysteine level in those losing weight) [25]. The carbohydrate restricted diet supplemented with folic acid in this study did not affect homocysteine concentrations. This is consistent with other work [26] and a prior study we published showing that weight loss induced by a low-fat diet and a 400 μg folic acid supplement resulted in no effect on homocysteine [27]. Therefore, vitamin supplementation or emphasis on folic acid-rich foods may be an important component of a weight loss program to prevent increases in homocysteine.

Homocysteine may be elevated following the ingestion of a high-protein meal even in individuals accustomed to higher protein intake. However, homocysteine levels tend to be normalized following fasting for an extended period of time (as in preparation for a blood draw) [28]. Postprandial measurements were not taken, though any potential increases in homocysteine appear to have been transient given the fasting plasma levels.

### Lp(a)

Elevated plasma Lp(a) is a risk factor for cardiovascular disease because of both atherogenic and thrombotic properties [4]. The 12% decrease in plasma Lp(a) is a novel finding considering that plasma Lp(a) levels are reported to have a strong genetic influence [29,30] and that diet usually has little positive influence on Lp(a) levels [5,31]. Important to note is that most diet interventions that have examined Lp(a) response emphasized fat restriction with moderate to high carbohydrate intake, which sharply contrasts the macronutrient contribution in the current intervention. Reducing total and saturated fat intake has been shown to increase Lp(a) during weight maintenance [32] and have no effect on Lp(a) during weight loss [33]. The significant decrease in Lp(a) in this study suggests that carbohydrate restriction, as opposed to fat restriction, may play a greater role in modulating Lp(a) levels during weight loss. Diets relatively high in trans fatty acids in comparison to saturated or unsaturated fatty acids can significantly increase plasma Lp(a) [34]. Thus, the 38% reduction in trans fatty acid consumption could also have also influenced this parameter.

Reports about weight loss and change in plasma Lp(a) are varied. Significant weight loss achieved via a very-low calorie diet in obese individuals has been shown to both reduce [35] and not reduce plasma Lp(a) [36]. However, a very-low calorie diet and weight loss may only reduce plasma Lp(a) in subjects with elevated baseline values (> 20 mg/dL). Our results are in agreement with previous reports that reductions in plasma Lp(a) correlate strongly with baseline Lp(a), but not with weight loss [36,37].

### CRP, IL-6, and TNF- α

Both the acute-phase reactant CRP and the inflammatory cytokine TNF-α were significantly reduced over time. These results extend our previous findings that weight loss achieved through carbohydrate restriction can improve plasma levels of inflammatory biomarkers [38] indicating that these results persist through 12 wk. Previous reports [38,39] suggest that weight loss – rather than the macronutrient composition of the diet used to achieve weight loss – is key to reducing plasma markers of inflammation. In the present study, a 7.9% weight loss was accompanied by an 18.5% reduction in CRP, which is consistent with other reports where low fat [40] and CRD [41] were used to achieve weight loss. We did not find a correlation between weight or fat loss and changes in any inflammatory marker. Change in CRP was significantly correlated with baseline levels, which is similar to a previous report utilizing a CRD [41]. Another positive alteration in inflammatory status was the reduction in TNF-α. Obese individuals release greater quantities of TNF-α, which is at least partially derived from adipose tissue [42]. The significant reductions in adipose tissue likely played a role in reducing plasma TNF-α. As such, it appears that carbohydrate restriction could be considered an option for overweight men to improve cardiovascular risk.

We also examined the individual data for CRP and TNF-α. The range for subjects with relative reductions in CRP (n = 21) was -2.9 to – 71.9%, and the range for subjects with relative increases in CRP (n = 8) was 1.5 to 43.0%. Fourteen of 21 subjects who reduced CRP had relative reductions greater than 20%. Five of eight subjects who had increases in CRP had elevations greater than 20%. Though the mean reductions and individual data indicate more individuals reduced CRP, some individuals had increases, indicating that weight loss from a CRD may not be universally appropriate for reducing CRP. However, CRP concentrations in individuals who experienced elevations after 12 wk was 1.36 mg/L (range = 0.14 to 4.17 mg/L), which is within the normal range. Similarly, 22 subjects had relative reductions in TNF-α. Our observations indicate that a CRD is generally effective at reducing both CRP and TNF-α, though, as with any dietary therapy, individual cases should be evaluated to determine appropriate treatment.

An unexpected result was that IL-6 was unchanged from baseline to week 12. IL-6 has often been reported to be reduced following weight loss [43,44] but not always [40,45]. IL-6 is thought to be important in the inflammatory cascade associated with atherogenesis because of its role in both stimulating several cellular adhesion molecules and smooth muscle cell proliferation and migration [42]. IL-6 also acts as a messenger cytokine [46] and that derived from adipose tissue is thought to provide the principle stimulus for hepatic CRP production [16,42]. Though a considerable amount of plasma IL-6 in healthy individuals is thought to be derived from adipose tissue (approximately 30%), a large proportion is derived from other tissue [47]. Thus, though weight loss – particularly fat loss – would be expected to reduce plasma IL-6, other influencing factors likely play an important role.

One potential tissue that may be implicated is skeletal muscle. It has been reported that consuming a CRD leads to an approximate 50% reduction in skeletal muscle glycogen [48], which could potentially affect plasma IL-6 levels. Steensberg et al. [49] hypothesized that contracting skeletal muscle releases IL-6, which acts in a hormone-like manner to stimulate hepatic glucose output. Subsequent work indicated that IL-6 release from skeletal muscle is increased in response to reduced glycogen levels [50]. It is important to note that reduction of glycogen was accomplished through exercise rather that diet. However, the response of IL-6 to reduced glycogen, taken in conjunction with observations that infusion of recombinant IL-6 in humans increases liver glucose output [50,51], suggest a potential explanation for the unchanged IL-6 levels (despite weight loss) in the present study. Future research is needed to confirm the role of IL-6 in glycogen depletion resulting from a CRD.

CRD have been consistently shown to reduce triglycerides and increase HDL-C, yielding a beneficial effect on CVD risk, particularly for individuals with metabolic syndrome [52]. For the current study we correlated triglyceride and HDL-C responses with emerging risk factors to determine if plasma lipid responses were related to changes in other risk factors for CVD. We found no relationship between response of traditional markers for CVD (HDL-C and triglyceride response) and relative changes in TNF-α, IL-6, CRP, Lp(a), and homocysteine. As such, we believe that individuals who have a less favorable triglyceride and HDL-C response to a CRD do not necessarily have an inferior response in terms of CVD risk markers not traditionally measured.

## Conclusion

We have previously reported the beneficial effects of a CRD on fasting and postprandial plasma lipoproteins and body composition. Data from this study indicate that the beneficial effects of a CRD on cardiovascular risk extend beyond improvements in traditionally measured markers, and that changes in these emerging biomarkers [Lp(a), CRP, TNF-α] are not related to the relative response in plasma triglycerides or HDL-C. These results are limited to the population of overweight or slightly obese men who are otherwise healthy. We cannot isolate the specific aspect of the diet responsible for the responses in emerging biomarkers, nor can we determine the relative contribution of weight loss versus carbohydrate restriction. An important finding was that carbohydrate restriction resulted in a spontaneous decrease in caloric intake and subsequent weight loss and improvement in several risk factors for cardiovascular disease in the majority of subjects. Thus, it can be concluded that a diet approach based on carbohydrate restriction provides an acceptable and effective model diet to combat obesity and related metabolic disorders.

## Abbreviations

BMI, body-mass index; CR, carbohydrate restriction; CRD, carbohydrate restricted diet; CRP, C-reactive protein; ELISA, enzyme-linked immunoassay; HDL-C, HDL-cholesterol; IL-6, interleukin 6; LDL-C, LDL-cholesterol; Lp(a), lipoprotein (a); TNF-α, tumor necrosis factor alpha.

## Competing interests

The author(s) declare that they have no competing interests.

## Authors' contributions

RJW was responsible for data acquisition, analysis, and interpretation and for drafting of the manuscript. JSV was responsible for study conception and design, data collection, analysis and interpretation and for critical revision of the manuscript. SRD was responsible for data analysis and interpretation and critical revision of the manuscript. CD was responsible for data analysis and interpretation and critical revision of the manuscript. MLF was responsible for study conception and design, data collection, analysis and interpretation, and for critical revision of the manuscript.
